# Beyond the Rash: Cutaneous Peripheral T-Cell Lymphoma as a Diagnostic Pitfall in Primary Care

**DOI:** 10.7759/cureus.102378

**Published:** 2026-01-27

**Authors:** Jared Hensley, Aura Calderon, Blake C Martin, Cristina Kochi, Andres R Suarez Parraga

**Affiliations:** 1 Internal Medicine, The University of Texas Rio Grande Valley School of Medicine, Edinburg, USA

**Keywords:** cutaneous t-cell lymphoma, goals of care, hematologic malignancy, inpatient medicine, rash differential diagnosis

## Abstract

Cutaneous manifestations of T-cell lymphomas may resemble common dermatologic or infectious conditions, increasing the risk of delayed recognition. We report a 78-year-old woman with known peripheral T-cell lymphoma (PTCL) who presented with a rapidly progressive, diffuse erythematous rash accompanied by cervical lymphadenopathy. Initial evaluation prioritized more common etiologies; however, inpatient medicine broadened the differential diagnosis, prompting early hematology and oncology consultation. Contrast-enhanced CT of the neck demonstrated multiple enlarged, partially necrotic left cervical lymph nodes consistent with disease progression. After multidisciplinary discussions and consideration of prognosis, the patient elected palliative-focused management with oncology follow-up. This case underscores the importance of emergency and primary care clinicians to include cutaneous lymphoma in the differential diagnosis of new diffuse rashes in patients with known or suspected hematologic malignancy, particularly when accompanied by lymphadenopathy or rapid progression.

## Introduction

Cutaneous T-cell lymphomas (CTCL) are uncommon extranodal non-Hodgkin lymphomas defined by malignant, skin-homing T cells and account for fewer than 4% of non-Hodgkin lymphomas in Western populations [1]. The most frequent subtypes are mycosis fungoides and Sézary syndrome; however, systemic peripheral T-cell lymphoma (PTCL) may also involve the skin and present with new or progressive cutaneous findings [2,3].

For first-contact clinicians, recognition is challenging because CTCL and secondary cutaneous involvement from PTCL can mimic common conditions, such as eczema, drug eruptions, or allergic hypersensitivity reactions [4,5]. As a result, diffuse pruritic rashes are often initially managed as benign inflammatory or allergic processes, particularly in emergency and primary care settings where immediate stabilization and common diagnoses appropriately dominate early triage. In patients with known or suspected hematologic malignancy, accompanying red flags, such as constitutional symptoms, lymphadenopathy, or rapid progression, should prompt a broadened differential that includes cutaneous lymphoma and early specialty consultation. In this case, an initial hypersensitivity-focused impression shifted to a malignancy-centered differential after inpatient reassessment in the context of prior PTCL and exam findings, leading to expedited hematology and oncology involvement.

## Case presentation

Patient information

A 78-year-old woman with a history of PTCL diagnosed in 2023, cardiac arrhythmias status post pacemaker-defibrillator placement, peripheral arterial disease, heart failure with reduced ejection fraction, obesity (body mass index approximately 31), and type 2 diabetes mellitus presented in August 2025 with a two-day history of a pruritic rash.

Clinical findings

**Figure 1:**
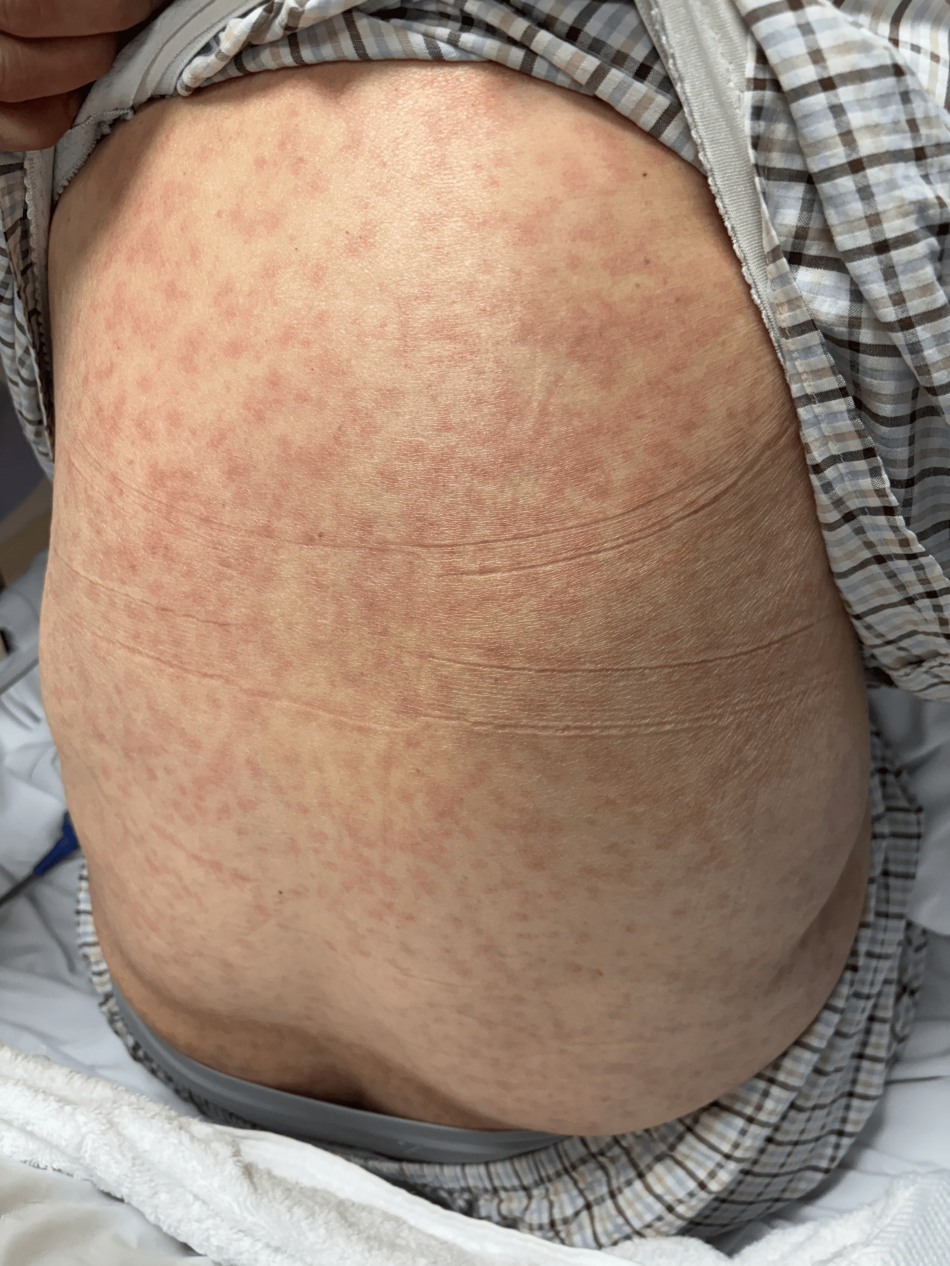
Diffuse erythematous patches and plaques involving the posterior trunk, demonstrating widespread truncal involvement.

**Figure 2:**
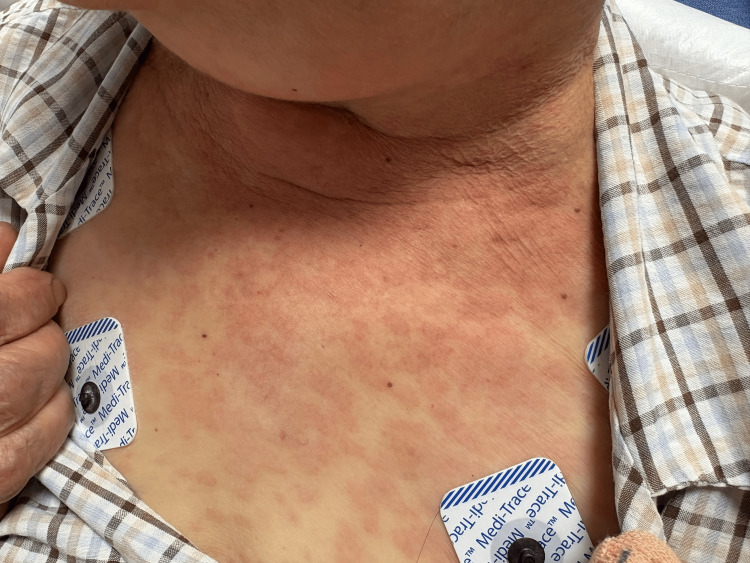
Scattered erythematous papules and plaques on the anterior chest.

**Figure 3:**
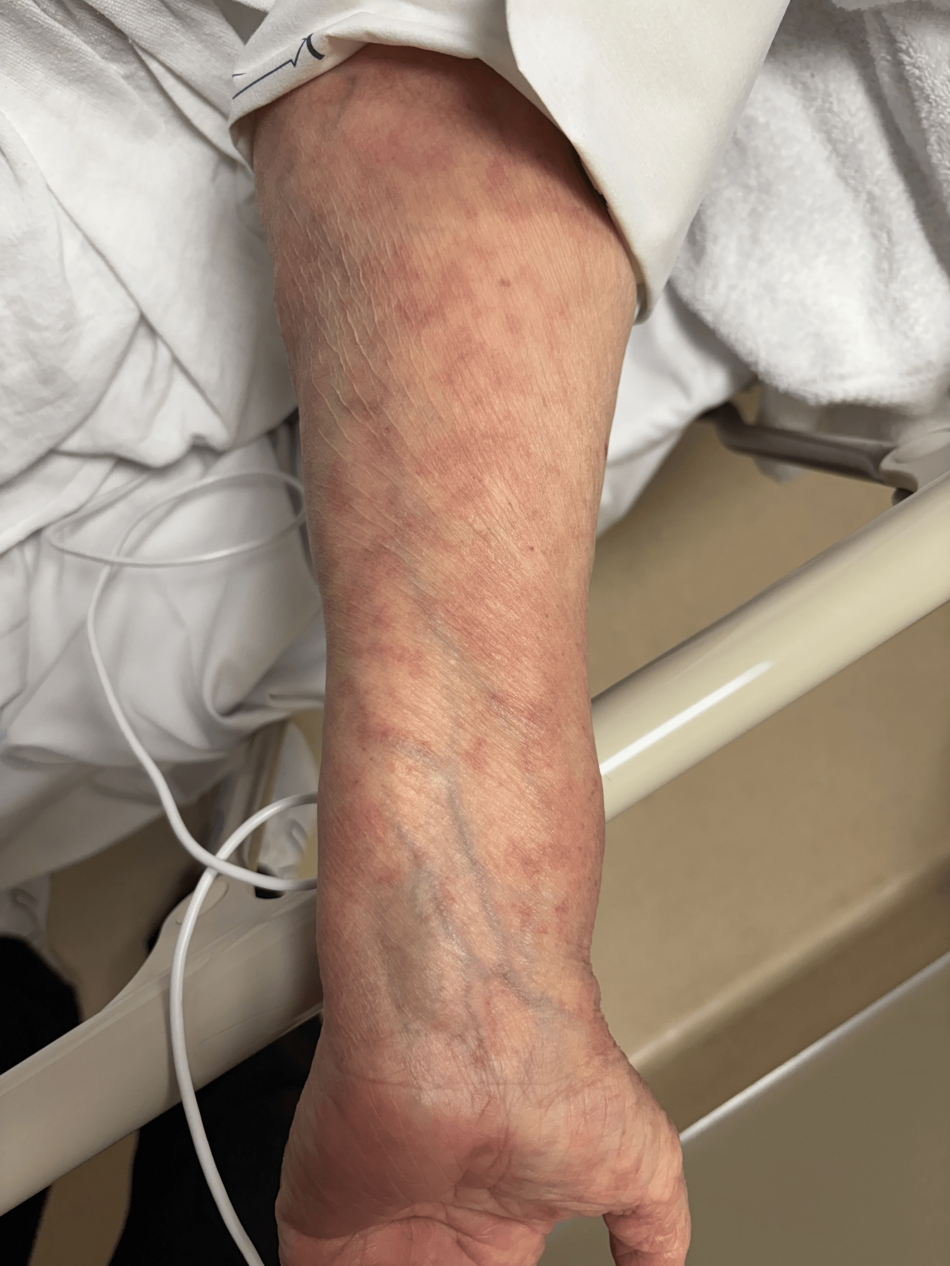
Erythematous plaques on the forearm, with mild surface scale.

The patient reported rapid progression of a pruritic rash from the bilateral forearms to the back and flanks. She denied fever, chest pain, dyspnea, or mucosal symptoms. Physical examination revealed numerous erythematous, slightly elevated patches and plaques involving the upper extremities and trunk, with relative sparing of the palms and soles. Mild left cervical lymphadenopathy was appreciated. No oral or ocular mucosal lesions were observed. Representative clinical photographs are shown in Figures 1-3. Written consent for clinical photography was obtained.

Diagnostic assessment

Initial laboratory evaluation demonstrated mild normocytic anemia and relative lymphopenia without leukocytosis (Table 1). Hemoglobin level was 11.4 g/dL, with a normal mean corpuscular volume, while the absolute lymphocyte count was reduced to 0.44 × 10⁹/L, findings that may be seen in advanced lymphoproliferative disorders. The differential showed neutrophil predominance without eosinophilia, making an allergic or hypersensitivity-mediated process less likely.

**Table 1:** Complete blood count on presentation. Reference ranges represent standard adult laboratory values (institution-specific ranges may vary).

Parameter	Result	Reference range
White blood cell count	6.9 × 10⁹/L	4.0-11.0 × 10⁹/L
Red blood cell count	3.76 × 10¹²/L	3.9-5.2 × 10¹²/L
Hemoglobin	11.4 g/dL	12.0-16.0 g/dL
Hematocrit	34.3%	36%-46%
Mean corpuscular volume	91.2 fL	80-100 fL
Platelet count	180 × 10⁹/L	150-400 × 10⁹/L
Neutrophils	88.3%	40%-70%
Lymphocytes	6.4%	20-45%
Absolute neutrophil count	6.11 × 10⁹/L	1.5-7.5 × 10⁹/L
Absolute lymphocyte count	0.44 × 10⁹/L	1.0-4.0 × 10⁹/L
Eosinophils	0.1%	0%-6%

Serum chemistry testing revealed preserved renal function with mild hyperchloremia and marked hyperglycemia, as well as hypoalbuminemia (Table 2). The low albumin level likely reflected chronic disease burden and systemic inflammation rather than acute hepatic dysfunction, as transaminases and bilirubin were within normal limits.

**Table 2:** Serum chemistry results on presentation. Reference ranges represent standard adult laboratory values (institution-specific ranges may vary).

Parameter	Result	Reference range
Sodium	143 mmol/L	135-145 mmol/L
Potassium	3.6 mmol/L	3.5-5.0 mmol/L
Chloride	111 mmol/L	98-107 mmol/L
Carbon dioxide (bicarbonate)	25 mmol/L	22-29 mmol/L
Blood urea nitrogen	19 mg/dL	7-20 mg/dL
Creatinine	0.82 mg/dL	0.5-1.1 mg/dL
Estimated glomerular filtration rate (GFR)	73.17 mL/min/1.73 m²	>60 mL/min/1.73 m²
Glucose	295 mg/dL	70-99 mg/dL
Calcium	9.2 mg/dL	8.5-10.5 mg/dL
Total bilirubin	0.8 mg/dL	0.2-1.2 mg/dL
Aspartate aminotransferase (AST)	18 U/L	10-40 U/L
Alanine aminotransferase (ALT)	27 U/L	7-56 U/L
Alkaline phosphatase	41 U/L	44-147 U/L
Total protein	6.0 g/dL	6.4-8.3 g/dL
Albumin	2.8 g/dL	3.5-5.0 g/dL

Urinalysis demonstrated bacteriuria with pyuria and positive nitrites in the absence of urinary symptoms (Table 3), consistent with asymptomatic bacteriuria rather than an active urinary tract infection. This finding was not felt to explain the patient’s cutaneous or systemic presentation.

**Table 3:** Urinalysis results. Reference ranges represent standard adult laboratory values (institution-specific ranges may vary). HPF: high-power field.

Parameter	Result	Reference range
Urine color	Dark yellow	Yellow
Urine appearance	Turbid	Clear
Urine pH	5.5	5.0-8.0
Specific gravity	1.021	1.005-1.030
Protein	30 mg/dL	Negative
Glucose	Normal	Negative
Blood	Large	Negative
Nitrite	Positive	Negative
Bilirubin	Negative	Negative
Urobilinogen	Normal	Normal
Leukocyte esterase	250	Negative
Red blood cells	10-25/HPF	0-3/HPF
White blood cells	26-50/HPF	0-5/HPF
Epithelial cells	1+	None/rare
Bacteria	4+	None
Hyaline casts	0-2	0-2
Mucus	3+	None/rare

**Figure 4:**
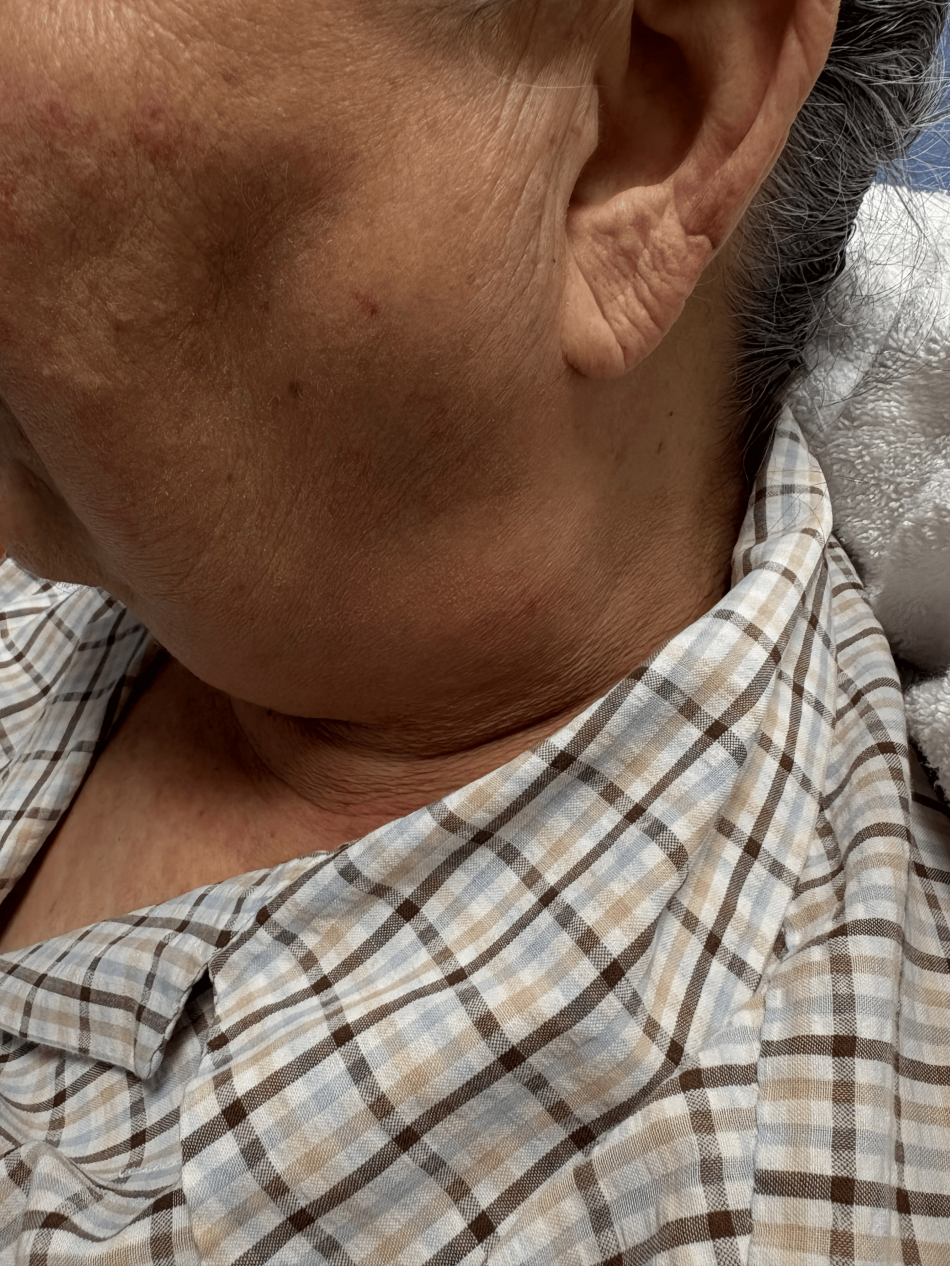
Visible fullness of the left parotid region on physical examination, corresponding to underlying cervical adenopathy.

**Figure 5:**
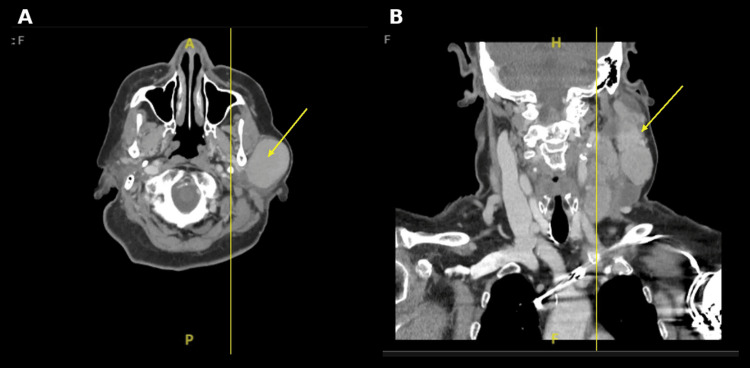
Contrast-enhanced computed tomography of the neck, demonstrating left cervical lymphadenopathy. (A) Axial view showing an enlarged, heterogeneous left cervical lymph node (arrow). (B) Coronal reconstruction demonstrating bulky left cervical adenopathy (arrow), most prominent at levels II and III. Multiple enlarged lymph nodes measured up to 4.3 cm with features concerning necrosis.

On physical examination, visible fullness of the left parotid region was noted, consistent with underlying lymphadenopathy (Figure 4). CT of the neck with intravenous contrast demonstrated multiple significantly enlarged left cervical lymph nodes, most prominent at levels II and III, measuring up to 4.3 cm (Figure 5A, 5B). Several lymph nodes exhibited features concerning necrosis. Associated enlargement of left parotid lymph nodes was present, with distortion of the left parotid and left submandibular glands by adenopathy, while the right parotid and submandibular glands appeared unremarkable. Mild asymmetric thickening of the left pharyngeal wall extending toward the left piriform sinus was observed. The airway remained patent.

When considered alongside the patient’s known history of PTCL and rapidly progressive cutaneous findings, these clinical and imaging findings raised concern for disease progression with secondary cutaneous involvement rather than an isolated hypersensitivity reaction.

Hospital course and management

In the emergency department, the patient was initially evaluated for an acute hypersensitivity or inflammatory eruption and treated with intravenous diphenhydramine and dexamethasone. Given the extent of the rash and her known history of PTCL, she was admitted to the inpatient internal medicine service for further evaluation. On admission, she was started on oral prednisone 40 mg daily and loratadine 10 mg daily.

Following inpatient reassessment, the differential diagnosis was broadened beyond hypersensitivity reactions to include malignancy-related etiologies in light of her prior PTCL diagnosis, cervical lymphadenopathy, and visible left parotid region mass. Hematology and oncology were consulted, and contrast-enhanced CT imaging of the neck demonstrated multiple enlarged left cervical lymph nodes with partial necrosis, consistent with disease progression. Blood cultures remained negative. Although empiric ceftriaxone was initiated after urinalysis revealed bacteriuria, antibiotics were discontinued when the patient remained asymptomatic, consistent with antimicrobial stewardship guidelines [6].

The patient’s rash improved during hospitalization without evidence of airway compromise, and she tolerated oral intake without difficulty. A prednisone taper was prescribed at discharge. During goals-of-care discussions involving the patient and her family, she expressed willingness to pursue palliative-directed chemotherapy after previously declining systemic treatment. She was discharged home with close oncology follow-up arranged.

Differential diagnosis

Given the patient’s acute, diffuse pruritic eruption in the setting of known PTCL and concurrent cervical lymphadenopathy, the differential diagnosis included malignant cutaneous involvement and common inflammatory mimics.

Secondary Cutaneous Involvement by PTCL

Secondary cutaneous involvement was strongly favored due to the patient’s established systemic PTCL, rapid onset of widespread plaques and patches, and evidence of progressive cervical nodal disease on examination and CT imaging.

Primary Cutaneous T-Cell Lymphoma

Primary CTCL was considered but less likely. Mycosis fungoides typically follows an indolent course, and Sézary syndrome presents with erythroderma and systemic involvement, including circulating malignant cells. The patient’s prior systemic PTCL and abrupt tempo argued against a primary cutaneous process.

Drug Eruption

Drug eruption was considered but less supported because the patient denied recent initiation of new medications, and the morphology was not clearly morbilliform.

Hypersensitivity Reaction

Hypersensitivity reaction was initially favored given the acute pruritic presentation and partial symptomatic improvement with antihistamines and corticosteroids; however, the lymphadenopathy and malignancy history suggested this diagnosis alone was insufficient.

Psoriasis

Psoriasis was considered due to plaque-like lesions, but the distribution, limited scale, and sudden onset were not typical.

## Discussion

Cutaneous involvement by T-cell lymphomas can present with nonspecific morphologies that overlap with common benign eruptions, including eczematous dermatitis, drug reactions, and urticaria, contributing to delayed recognition [1,4,7]. This diagnostic ambiguity is especially consequential in first-contact settings, where acute pruritic rashes are often approached as hypersensitivity reactions. In our patient, the initial working diagnosis prioritized common etiologies; however, her known PTCL, associated cervical lymphadenopathy, and rapid eruption progression prompted inpatient reassessment and early hematology/oncology involvement, reframing the presentation as likely disease progression rather than an isolated allergic process.

Although classic primary CTCLs, such as mycosis fungoides and Sézary syndrome, are staged using the TNMB system, systemic lymphomas and advanced disease can involve the skin and mimic inflammatory dermatoses [1,2]. Consensus frameworks emphasize that assessment of CTCL-spectrum disease requires integration of cutaneous findings with systemic features and longitudinal clinical context, as single time-point evaluation may be misleading [3,8]. Importantly, symptomatic improvement with corticosteroids or antihistamines does not exclude malignancy and may transiently blunt visible inflammation, increasing the risk of diagnostic anchoring in patients with underlying hematologic disease [4]. In patients with known or suspected hematologic malignancy, a new diffuse eruption accompanied by systemic symptoms, lymphadenopathy, or adjacent glandular masses should prompt a broadened differential and early specialist involvement, with imaging and biopsy pursued when feasible [1,4,7].

Population-based data underscore the rarity of CTCL, which contributes to limited clinician familiarity and a lower pretest probability outside specialty settings [9]. This reinforces a practical approach for primary care and emergency clinicians: when a patient has a history of lymphoma (or concerning systemic findings), the threshold to reconsider common diagnoses and escalate evaluation should be lower than it would be for an otherwise healthy patient with an isolated rash.

This case also highlights a management inflection point that is common in advanced malignancy but underemphasized in case reports: inpatient discussions can meaningfully change treatment trajectories. The patient had previously declined therapy; however, after multidisciplinary conversations that included the patient and family, she elected a palliative-focused approach with oncology follow-up. For primary care and emergency clinicians, the key takeaway is twofold: maintain diagnostic vigilance for lymphoma-related cutaneous disease when red-flag features are present, and recognize that clear, patient-centered communication about prognosis and options can be as clinically consequential as the diagnostic workup itself.

## Conclusions

Clinicians in emergency and primary care settings should maintain a high index of suspicion for cutaneous lymphoma when evaluating new or rapidly progressive diffuse rashes in patients with known or suspected hematologic malignancy, particularly when accompanied by lymphadenopathy or other red-flag features. This case illustrates how anchoring on common inflammatory diagnoses can delay recognition of malignancy-related disease, even when initial symptomatic improvement is observed. Equally important, timely inpatient reassessment created an opportunity for meaningful goals-of-care discussions, ultimately guiding the patient toward palliative-directed management aligned with her values. Together, these findings highlight the dual importance of diagnostic vigilance and patient-centered communication in the care of individuals with complex oncologic disease.
